# Wrap It! Preventive Antimicrobial Treatment Shows No Negative Effects on Tenocytes and Tendons—A Comprehensive Approach

**DOI:** 10.3390/jcm12124104

**Published:** 2023-06-17

**Authors:** Manuela Thierbach, Michelle Müller, Richard Stange, Daniel Kronenberg, Matthias Aurich, Britt Wildemann

**Affiliations:** 1 Experimental Trauma Surgery, Department of Trauma, Hand and Reconstructive Surgery, Jena University Hospital/Friedrich Schiller University Jena, 07747 Jena, Germany; manuela.thierbach@med.uni-jena.de (M.T.); michelle.mueller@med.uni-jena.de (M.M.); matthias.aurich@uk-halle.de (M.A.); 2 Department of Regenerative Musculoskeletal Medicine, Institute for Musculoskeletal Medicine, Westfälische Wilhelms-Universität Münster, 48149 Münster, Germany; richard.stange@ukmuenster.de (R.S.); daniel.kronenberg@ukmuenster.de (D.K.); 3 DOUW—Section of Trauma and Reconstructive Surgery, University Hospital Halle (Saale), 06120 Halle, Germany; 4 BG Trauma Center Bergmannstrost, 06112 Halle, Germany

**Keywords:** ACL reconstruction, infection, vancomycin, wrap, soaking, tendon, tenocytes

## Abstract

Although the rate of infection after the reconstruction of a ruptured anterior cruciate ligament (ACL) is low, prophylactic incubation of the graft with vancomycin (Vanco-wrap or vancomycin soaking) is routinely performed. A cytotoxic effect of vancomycin is reported for several cell types, and the prophylactic treatment might prevent infection but harm the tissue and cells. Aim: A comprehensive study was performed to investigate the effect of vancomycin on tendon tissue and isolated tenocytes using cell viability, molecular and mechanical analysis. Material and methods: Rat tendons or isolated tenocytes were incubated in increasing concentrations of vancomycin (0–10 mg/mL) for different times, and cell viability, gene expression, histology and Young’s modulus were analyzed. Results: The clinically used concentration of vancomycin (5 mg/mL for 20 min) had no negative effect on cell viability in the tendons or the isolated tenocytes, while incubation with the toxic control significantly reduced cell viability. Increasing the concentration and prolonging the incubation time had no negative effect on the cells. The expression of *Col1a1*, *Col3a1* and the tenocyte markers *mohawk*, *scleraxis* and *tenomodulin* was not affected by the various vancomycin concentrations. The structural integrity as measured through histological and mechanical testing was not compromised. Conclusion: The results proved the safe application of the Vanco-wrap on tendon tissue. Level of evidence: IV.

## 1. Introduction

The anterior cruciate ligament (ACL) is one of the most frequently injured ligaments [1,2] and ACL reconstruction (ACLR) is one of the three most commonly performed surgeries in orthopedics, and different techniques are used for this [3,4,5,6]. In Germany, the annual incidence of an ACL injury amounts to 40–50 cases per 100,000 citizens [7]. Based on existing register and insurance data, the incidence of ACLR is assumed to be between 29 and 38 per 100,000 people [8]. Because of its poor biological ability to heal, the ACL is generally reconstructed using an autograft from either the hamstrings or patella tendon [9]. The preparation of the graft bears the risk of contamination and a small study showed that up to 13% of the analyzed ACLRs were positive for bacteria, though no infection was observed postoperatively [10]. However, infection after ACLR is a feared complication and the analysis of 1564 ACLR cases revealed an infection rate of 0.45%, where coagulase-negative Staphylococcus species have been the most frequent pathogens, followed by *Enterococcus faecalis* and *Enterobacter cloacae* [11]. Knee joint infections with Staphylococcus species occur mostly as a result of contamination from patient’s skin during graft preparation, as these bacteria belong to the normal skin microbiome [12]. A recent study investigated the contamination of allografts for ACLR during operation/suturing and demonstrated a high contamination rate after graft preparation (21.9%) [13]. This is in accordance with studies showing that prolonged operation time is clearly associated with a higher risk of infection [14,15]. Bacterial contamination during surgery was detected on surgical devices, the operation field, sucker tips and the collection bag [16].

To prevent septic arthritis after ACLR, the harvested autograft can be wrapped in a sterile gauze swab soaked in vancomycin before reinsertion [17]. A meta-analysis including 68,453 grafts found infections after ACLR in 0.9% of patients, where only 0.1% of infections were seen in patients with Vanco-wrap [18]. A smaller meta-analysis (5075 patients) found infection rates of 2.1%, with a reduction to 0% for Vanco-wrap [19].

Vancomycin is a glycopeptide antibiotic effective against almost all Gram-positive bacteria [20]. The bactericidal effect unfolds through the inhibition of the bacterial cell wall synthesis. Since its discovery in the 1950s, vancomycin has become widely used because of its excellent activity against both *Clostridium difficile* in pseudomembranous enterocolitis and methicillin-resistant *Staphylococcus aureus* (MRSA) [21]. Regardless of its beneficial effects, vancomycin is also known for having cytotoxic effects on mesenchymal stromal cells and osteoblasts, as well as chondrocytes [22,23,24,25]. A meta-analysis on the effect of intrawound vancomycin powder application (1–2 g) in knee and hip arthroplasty revealed an effective infection prophylaxis but an increase in aseptic wound complications [26], raising concerns about the healing process. Previously, studies have been conducted to investigate the effects of vancomycin on ACL grafts. Tendons incubated with vancomycin serve as a reservoir and elute the antibiotic over a period of up to 24 h depending on the graft size and incubation time [27]. Schüttler et al. showed successful eradication of *S. epidermidis* without affecting the mechanical properties of the porcine flexor tendon after vancomycin incubation [28]. Studies regarding a possible cytotoxic effect on tenocytes are controversial regarding the safe vancomycin concentration [29,30,31].

It is known that various factors may influence tendon biology, structure, metabolism and functional parameters, which also interact with each other in a complex manner. However, until now, no study combined in vitro tissue and cell viability testing, biomechanical and histological analysis as well as molecular studies to reflect these interactions in the context of vancomycin application. Therefore, in the present study, we investigated variations in vancomycin concentration, duration and timing of aftereffects on tendons for the first time in a comprehensive approach using tendons from rats of both sexes and different breeds and ages to reflect different clinical situations.

We hypothesized that no negative effects of the vancomycin wrap as used in the clinical setting would be seen in the biological and mechanical analysis, but the higher concentrations and durations might harm the tissue and cells. The purpose of the present work was, therefore, to investigate possible negative effects of different vancomycin concentrations and incubation durations on tendons and isolated tenocytes in vitro. Such a multifaceted approach using standardized tissue samples from rats can also be helpful in other tendon and ligament research areas for testing the effects of drugs or treatments on tendons, ligaments and cells.

## 2. Materials and Methods

### 2.1. Animal Tissue Samples

In accordance with the 3R principle (replace, reduce, refine), surplus Achilles tendons were obtained after the animals were used in other experiments, which were approved by local authorities (Thüringer Landesamt für Verbraucherschutz). To reflect the clinical situation, rats of both sexes, different breeds and ages ranging from 10–80 weeks were used. The animals had no musculoskeletal or other injuries or diseases. The rats were housed under standard conditions with a temperature-controlled environment, 12 h light/dark cycle and unlimited access to food (Normal Chow: V1534 with 9 kJ% fat, 24 kJ% protein and 67 kJ% carbohydrates) and drinking water. Deep anesthesia was induced using thiopental (150 mg/kg bodyweight) before animals were sacrificed. Left and right Achilles tendons from each rat were dissected, and the surrounding tissue, bone and muscle were eliminated carefully. For the Vanco-wrap experiment, the tendons were divided longitudinally into three similar sections. For tenocyte isolation, the whole tendon was used.

### 2.2. Tenocyte Isolation

Achilles tendons were washed in PBS, minced into small pieces and digested with Collagenase-P (Roche Diagnostics GmbH, Mannheim, Germany) in DMEM for 2.5 h at 37 °C. After centrifugation, removing digestion medium and resuspending the pellet, the cells were cultured in DMEM + 10% FCS + 1% Pen/Strep at 37 °C with 95% air and 5% CO_2_. Tenocytes were harvested after 4 days, counted and stored at −150 °C in freezing medium with DMSO.

### 2.3. Vancomycin Dilution

For the cell culture experiment with isolated tenocytes, vancomycin (Vancomycin CP 1.0 g, Hikma Pharma GmbH, Martinsried, Germany) was pre-dissolved in physiological saline, and solutions of different concentrations ranging from 0.0125 mg/mL up to 10 mg/mL were prepared in cell culture medium DMEM (PAN Biotech, Aidenbach, Germany) + 10% FCS + 1% Pen/Strep. For the wrap experiment, vancomycin solutions of 5 mg/mL and 10 mg/mL were prepared by dissolving in physiological saline.

### 2.4. Vanco-Wrap with Tendon Samples

Before (and after) incubation, the tendon tissue samples were rinsed in PBS and transferred into 48-well plates for cell viability testing (see below). Then, the tendons were placed into sterile fleece saturated with vancomycin solution of 5 mg/mL and 10 mg/mL for 10, 20 or 40 min at RT. Tendon samples in NaCl, EtOH and medium-saturated fleece served as controls. After wrap incubation, half of the tendon was used for live/dead assay or for HE staining (see below). With the other half, gene expression analysis was performed; after snap-freezing in liquid nitrogen and storing at −80 °C, RNA isolation was carried out. A sample size of *n* = 9 for 5 mg/mL or 11 for 10 mg/mL vancomycin was used.

### 2.5. Tenocyte Culture and Treatment with Vancomycin

Tenocytes were thawed and 2.4 × 10^3^ cells per well were cultured in a 48-well plate overnight to establish cell attachment as described previously [32,33]. Baseline cell viability was detected at day 0 and cells were incubated with vancomycin solutions from 0.0125 mg/mL up to 10 mg/mL for 20 min. Further cell viability tests were performed on day 1, 3 and 7, each with 3 technical replicates. On day 1 and 7, cells were harvested and stored at −80 °C for further qRT-PCR. A sample size of *n* = 5 was used.

### 2.6. Cell Viability

Cell viability before and after treatment with vancomycin was measured using the PrestoBlue Cell Viability Reagent (Invitrogen/Life Technologies Europe BV, Bleiswijk, The Netherlands) according to the manufacturer’s instructions. PrestoBlue is a very sensitive assay allowing the detection of low cell numbers, 10 cells/well, depending on the incubation time with a large dynamic range. When added to living cells or tissue, the PrestoBlue reagent is modified by the reducing environment of the viable cell. After 1 h, the resulting color change was detected using absorption measurement at 570 and 600 nm through microplate spectrophotometry (Biotek Epoch, Agilent Technologies, Waldbronn, Germany). Cell viability was related to tendon weight and tenocyte number.

### 2.7. Live/Dead Assay for Tendon Tissue

To discriminate live from dead cells, a LIVE/DEAD™ Viability/Cytotoxicity Kit (Invitrogen/Life Technologies Europe BV, Bleiswijk, The Netherlands) was used. Simultaneous staining with green fluorescent calcein-AM to indicate intracellular esterase activity and red fluorescent ethidium homodimer-1 to indicate the loss of plasma membrane integrity was carried out according to the manufacturer’s instructions. After staining, the tissue was fixed in 4% paraformaldehyde solution (PFA) for 24 h until rinsing, automatic dehydration and paraffin embedding. Slices 5 µm thick were cut, and fluorescence microscopy was carried out with a Zeiss Axioplan 2 Imaging Fluorescence Microscope (Zeiss, Göttingen, Germany). Unfortunately, the green fluorescence of live cells was destroyed due to the further processing. Therefore, sections were counterstained with DAPI to visualize all cells.

### 2.8. Hematoxylin Eosin (HE) Staining and Image Analysis

After fixing in 4% PFA for 24 h and automatic paraffin embedding, slices 5 µm thick were cut and stained with Mayer’s hemalum solution (Merck KGaA, Darmstadt, Germany) and Eosin G 0.1% (Merck KGaA). Following microscopy, analysis was performed with ImageJ 1.52 p software (Wayne Rasband, National Institute of Health, Bethesda, MD, USA). To contrast the surrounding tissue, the tenocyte nuclei were selected manually with the freehand tool. The parameters cell number, area, perimeter, circularity and minor and major dimension of the nuclei were evaluated.

### 2.9. qRT-PCR

Tendon samples stored at −80 °C were homogenized with TRIzol (Thermo Fisher Scientific, Waltham, MA, USA) using a T25 digital ULTRA-TURRAX (IKA, Staufen, Germany). The addition of chloroform caused phase separation. RNA was extracted from the aqueous phase. The tendon RNA and the RNA of the tenocytes from cell culture experiments was purified with a RNeasy Plus Mini Kit (Qiagen, Hilden, Germany) according to the manufacturer’s instructions and quantified with a NanoDrop 2000 c spectrophotometer (Thermo Fisher Scientific, Waltham, MA, USA). Using 100 ng RNA, template cDNA synthesis was performed with cDNA Supermix (Quanta Bioscience, Gaithersburg, MD, USA). qRT-PCR was carried out with SYBR^®^Green SuperMix (Quanta Biosciences) and Rotor-Gene Q (Quiagen). All primer sequences except the *scleraxis* primer (ordered from Qiagen, Cat No.: QT01596028) were designed using Primer3 (Version 0.4.0) and NetPrimer software and produced by TIB Molbiol, Berlin, Germany; sequences are as described previously [34]. The *18s* rRNA gene was chosen as the housekeeping gene. All primers were tested for amplification efficiency, and the ΔCt method with efficiency correction was used to calculate the relative gene expression to the reference gene *18s* rRNA. The normalized expression was calculated using the efficiency-corrected equation [35]. The sample size was *n* = 4.

### 2.10. Biomechanical Testing

The right Achilles tendon was used as the control, while the left tendon was incubated in vancomycin with a concentration of 5 mg/mL for 20 min, with a sample size of *n* = 10 per group. The tendons were stored at −80 °C until biomechanical testing then thawed at room temperature. With the use of an axial test-bench LM1 (TA Instruments/ElectroForce, New Castle, DE, USA), a displacement-controlled elongation was performed as described previously [36]. Briefly, the calcaneus and the proximal site of the tendon were fixed, and specimens were transferred to a PBS-bath at room temperature. Tendon dimensions including length and width were measured with cameras from two angles. Tendons were stressed until failure in a displacement-controlled load-to-failure ramp. Force measurements were normalized to the tendon diameter to obtain the Young’s modulus. The static Young’s modulus was calculated from the linear elastic region of the load-to-failure curve.

### 2.11. Statistics

Statistical analysis was carried out with Prism 9 (GraphPad, San Diego, CA, USA). The Kruskal–Wallis non-parametric test followed by Dunn’s correction for multiple testing was used. A *p*-value of ≤0.05 was considered statistically significant. The graphs show all data points as median with interquartile range.

## 3. Results

In agreement with the 3R principle, tendons were harvested from rats used in other experiments. The rats differed in age, sex and strain but were not treated in a way that might affect the tendon tissue.

### 3.1. Effect of Vanco-Wrap on Tendon Tissue

To determine the effect of the Vanco-wrap on the tendon tissue, histological, biomechanical, viability and expression analyses were performed.

**Figure 1:**
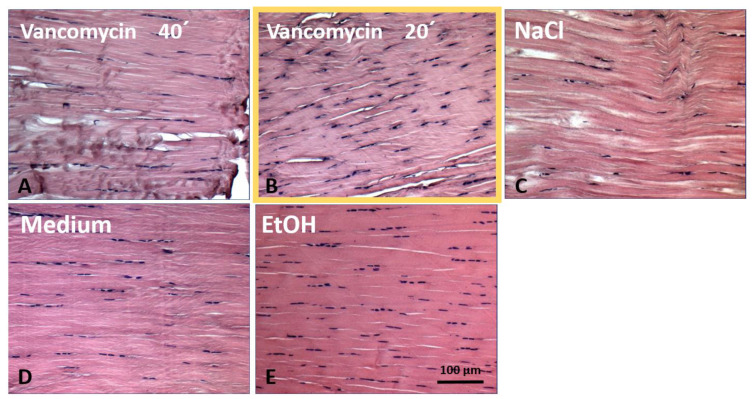
Hematoxylin eosin staining of tendon tissue. (**A**) After incubation with 5 mg/mL vancomycin for 40 min or (**B**) 20 min. (**C**,**D**) Controls incubated with sodium chloride (NaCl, dilution medium for vancomycin) or cell culture medium only. (**E**) Positive/cytotoxic control (EtOH, ethanol). No negative effect of the vancomycin treatment on the tissue structure was visible.

Incubation of the tendon tissue with vancomycin at different concentrations up to twice the clinical concentration and for 20 or 40 min had no effect on the tendon tissue, as shown by the histology (Figure 1). The incubation with ethanol (EtOH) as a cytotoxic agent also resulted in no obvious alterations of the tissue.

**Figure 2:**
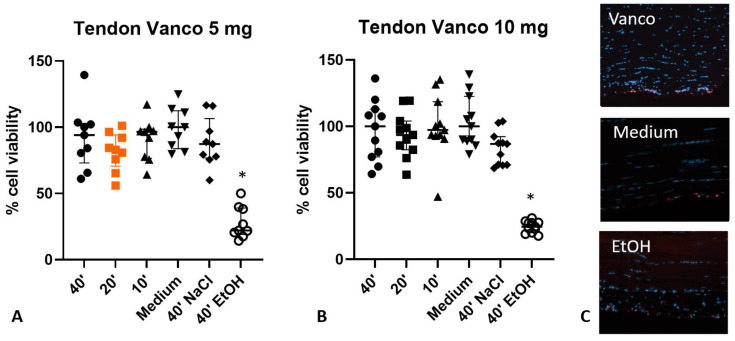
Cell viability in tendons after treatment with vancomycin (5 and 10 mg) for 10 to 40 min (**A**,**B**) (*n* = 9 or 11 per treatment). Only incubation with EtOH resulted in a significant reduction compared to medium-treated tendons. (**C**) Live/dead stain of the tissue. Blue: all cell nuclei, red: dead cells. Dead cells were mainly located at the surface of the tendon. Statistics: Kruskal–Wallis with Dunn’s multiple comparison to medium, * *p* < 0.0001 compared to medium. Orange indicates the clinical use. NaCl: sodium chloride; EtOH: ethanol.

Cell viability was assessed with the PrestoBlue assay, and no negative effect of the vancomycin incubation (5 or 10 mg) was detectable when compared to the control (40 min NaCl) (Figure 2).

Incubation of the tissue for 40 min with EtOH significantly reduced the cell viability in comparison to the medium control (*p* < 0.0001, Figure 2A,B). Staining of the tissue with ethidium homodimer-1 revealed dead cells only at the surface of the tendon and not within the tendon tissue (Figure 2C). Analyzing the parameters cell number, area, perimeter, circularity and minor and major dimension of the nuclei after hematoxylin eosin staining, no significant differences were detected in the samples treated for 20 min with 5 mg vancomycin, medium, NaCl or EtOH.

**Figure 3:**
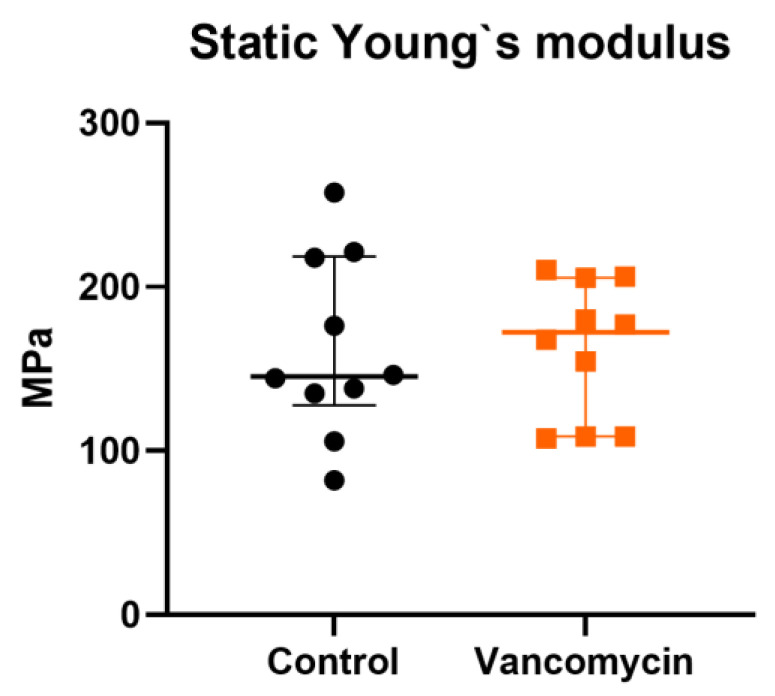
Biomechanical testing of the tendons after incubation with vancomycin. No significant difference (*p* = 0.8534) was detectable in the static Young’s modulus. Orange indicates the clinical use (5 mg/mL vancomycin for 20 min) (*n* = 10 per treatment). MPa: megaPascal.

Biomechanical elongation testing was performed to detect a possible effect of the vancomycin incubation on the mechanical properties of the tendon. The static Young’s-modulus revealed a median value of 145.3 (IQR 127.9–218.6) MPa for the control tendons and 172.4 (108.8–205.7) MPa for the vancomycin-treated tendons with no significant difference (Figure 3).

**Figure 4:**
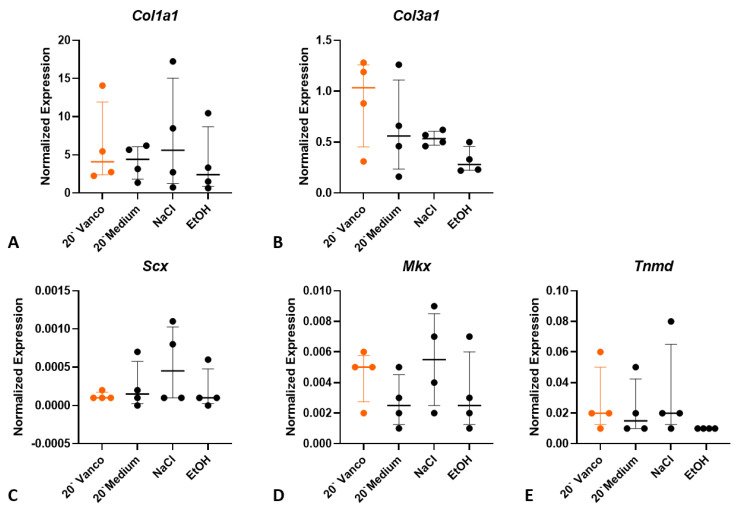
Expression analysis of collagens (**A**,**B**) and tenocyte markers (**C**–**E**) after incubation of the tendon with 5 mg/mL vancomycin for 20 min (clinical use) (*n* = 4 per treatment). Kruskal–Wallis with Dunn’s multiple comparison to medium, no significant differences. Orange indicates the clinical use. *Col: collagen*; *Scx: scleraxis*; *Mkx: mohawk*, *Tnmd: Tenomodulin*.

Even though no changes in cell viability were detected, a negative effect on gene expression might occur during incubation with vancomycin, which might alter the cell fate. The expression of the collagens *Col1α1* and *Col3α1* and the tenocyte markers *Scx*, *Mkx* and *Tnmd* were not significantly affected by vancomycin incubation for 20 min (Figure 4).

### 3.2. Effect of Vancomycin on Tenocytes

**Figure 5:**
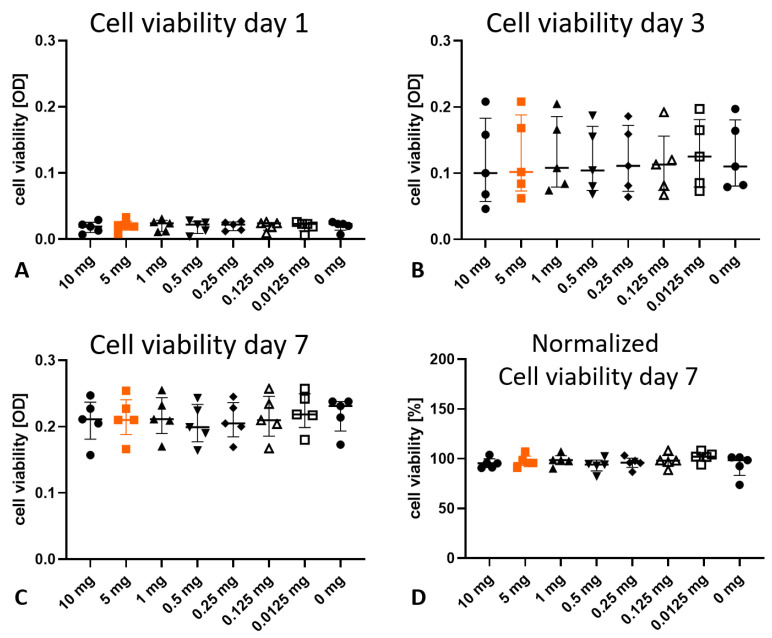
Cell viability of isolated tenocytes incubated with various concentrations of vancomycin for 20 min and cultured for a total of 7 days (*n* = 5 per treatment). (**A**–**C**) The cell viability increased over time in all groups without significant changes due to the treatment. (**D**) Normalizing the viability to the 0 mg vancomycin group (100%) revealed no negative effect of vancomycin. Orange indicates the clinical use.

The dense extracellular matrix of the tendon tissue might protect the tenocytes from the vancomycin. To investigate the possible effect directly on the cells, tenocytes were isolated and incubated with increasing concentrations of vancomycin (0–10 mg/mL) for 20 min and cultured for further 7 days. A clear increase in cell viability, as an indirect measure for cell number, was detected from day 0 to day 7 without differences between the groups on the individual days (Figure 5A–C).

Due to the inhomogeneous rat population used (age, sex, strain), the data have high variability. After normalization to 0 mg vancomycin on day 7, the data show a high level of homogeneity and no significant differences depending on the treatment (Figure 5D).

**Figure 6:**
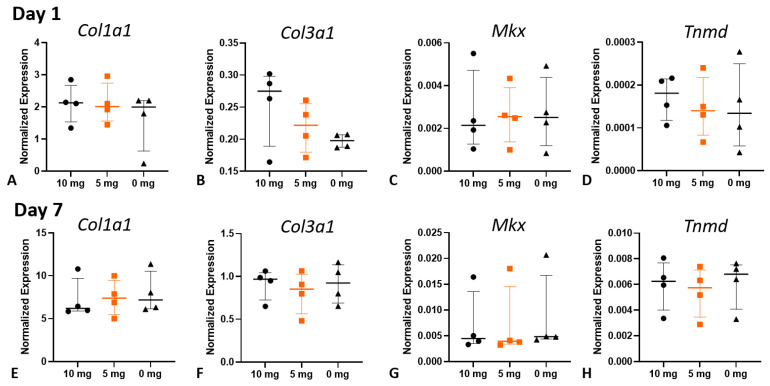
Gene expression data from the isolated tenocytes cultured for 1 (**A**–**D**) or 7 days (**E**–**H**) after incubation with vancomycin for 20 min (*n* = 4 per treatment). There were no significant changes due to the vancomycin treatment compared to the control (0 mg). Orange indicates the clinical use. *Col: collagen*; *Scx: scleraxis*; *Mkx: mohawk*, *Tnmd: Tenomodulin*.

RNA was isolated from the cell cultures on day 1 and day 7, and gene expression was analyzed (Figure 6). All investigated genes were expressed at higher levels on day 7 compared to day 1. The expression shows high variability and an increase in *Col3α1* after treatment with 10 mg vancomycin, though the effect was not significant (*p* = 0.3396 compared to 0 mg/mL).

## 4. Discussion

Infections after ACL reconstruction are rare complications, but can have devastating consequences for the patient and the treating surgeon. To kill bacteria that might colonize the transplant during harvesting and preparation, and to prevent further bacterial colonization of a transplant during storage until implantation, wrapping the transplant in a vancomycin-soaked gauze is frequently and effectively preformed [18,37]. The results of the present comprehensive study revealed no negative effect of the Vanco-wrap on tendon tissue or isolated tenocytes as determined using viability assays, mechanical testing and histological and molecular studies. Higher vancomycin concentrations, up to 10 mg, revealed no negative effects on cell viability and gene expression after a short incubation of 20 min, as is typically used in a clinical setting.

A study published in 2022 provided data on the cost-effectiveness of this procedure, with savings between EUR 226 in Spain and USD 660 in the USA [38]. The authors also determined that this treatment is cost-effective at very low infection rates higher than 0.014% (USA) or 0.02% (Spain). The cost-effectiveness is due to the low price of vancomycin and the simplicity of the procedure. Rodriguez-Merchan and Ribbans summarized the knowledge on the in vitro and in vivo studies performed on the vancomycin soaking procedure in their current concept review [39]. They concluded that, beside the published level III studies, prospective clinical trials are necessary to answer questions such as if this treatment should be used universally in all ACLRs, if incubation time might have an effect and if the vancomycin concentration might be adapted. However, not only efficacy matters; the vancomycin soaking should not harm the graft and should not have negative effects on ACLR. Clinical studies focused on this topic showed no effect on re-rupture rate and functional outcome [40,41,42]. However, it is well known that antibiotics and especially vancomycin can have dose-dependent negative effects on cells of the musculoskeletal system. Concentrations of >0.125 mg/mL for 48 h and longer have been shown to be cytotoxic for osteoblast-like cells and chondrocytes [24]. Testing the effect of vancomycin on different musculoskeletal cells, cytotoxic effects were reported at a low concentration of 0.01 mg/mL when cells were incubated with vancomycin for 3 days [43]. A dosage-dependent effect was also seen on mesenchymal stromal cells: the application of 4.8 mg/mL vancomycin for 24 h resulted in 13.58% cell death [44]. The present study aimed to use a comprehensive approach to investigate the effect of Vanco-wrap on tendon tissue and isolated tenocytes by assessing possible alterations through mechanical testing and histological and molecular analysis of tendon tissue and isolated cells. Using the clinical concentration and duration as standard, which is 5 mg/mL vancomycin for 20 min, we also tested different higher concentrations and longer incubation times up to 40 min. All experiments showed no negative effect of vancomycin on the tendon tissue or tenocytes, supporting the safety of this procedure. These non-cytotoxic effects seem to be in contrast to the previously reported results, but might be explained by the rather short incubation times of up to 40 min followed by a change to normal culture medium. The above-cited studies all used longer incubation times, which might be more toxic to the cells, but are not comparable to the clinical setting. Meanwhile, three further studies have been published investigating the effect of vancomycin on tenocytes. Atheron et al. used human hamstring tendons and investigated the effect of 5 mg/mL vancomycin on isolated tenocytes [30]. They found a slight but significant decrease in cell viability after 60 min of incubation with vancomycin but not after 30 and 120 min. The expression of apoptotic markers was not altered, but collagen1 and IL6 synthesis was significantly reduced. Papalia et al. investigated the effect of various vancomycin concentrations on human tenocytes and showed negative effects of vancomycin at concentrations higher than 2.5 mg/mL [29]. Cytotoxic effects with reduced cell viability and increased apoptosis were seen after 15 min incubation with 5 mg/mL and higher. For significant effects, however, incubation with 10 mg/mL for 60 min or 25 mg/mL for 10 min was necessary. Xiao et al. only saw a negative effect on the viability of isolated tenocytes after incubation with 6.4 mg/mL or higher for 24 h [31]. Using a rat ACLR model, a reduction in *S. aureus* contamination of grafts presoaked with 5 mg/mL vancomycin for 30 min was detected, without any influence on graft incorporation [45], whereas the rats with contaminated grafts and no vancomycin presoaking showed microbiological colonization 2 weeks post operatively, with radiological and histological signs of infection after 2 and 12 weeks. The biomechanical testing showed comparable values between the vancomycin-treated group and the native knee. Together with the results of the present study, the use of 5 mg/mL vancomycin for 20 min seems to be a safe and non-cytotoxic application. These results are in accordance with newer studies on the possible cytotoxicity of vancomycin on chondrocytes, showing no negative effect of 6.25 mg/mL at less than 9 days of incubation [23].

The analysis of the mechanical tendon properties after incubation with vancomycin for 20 min revealed no significant effect on the static Young’s modulus. This is in line with previous studies [28,46], in which researchers tested porcine or bovine tendons incubated for up to 30 min in vancomycin and detected no significant difference in the Young’s modulus between the groups. Using a rat ACLR model, Tong et al. found comparable mechanical properties of knees with vancomycin-treated tendons and intact knees 12 weeks after surgery [45], indicating no negative effect of the vancomycin treatment on the healed ACL.

In accordance with the 3R principle, we used tendons obtained from rats from other experiments. The rats differed in age (in a range from 10 to 80 weeks), sex and strain. The variations in the animals should resemble the variations in patients. The drawback of this approach is the higher variation we see in the data. However, the variations seen are comparable to studies using human samples [30].

Using the same conditions/treatments in a standardized set-up for the cells or tendons, a comparison of the vancomycin effect through the different methods is possible. Therefore, this is the first study showing no effect of vancomycin used in different concentrations on the viability of isolated cells or cells in the tendon, or on histological appearance, biomechanical properties and the expression of tendon-related markers.

As a limitation, the use of animal tissue should be mentioned. Due to the smaller size of the tissue and the higher surface area to volume ratio, the tissue and cells might be more prone to cytotoxic effects of vancomycin. The live/dead assay might have a methodological limitation when used with the tendon tissue. We cannot prove a sufficient penetration of the ethidium deep into the tissue, meaning our results could be false negatives for the tendon core. However, this will be the same for the toxic control (EtOH), where we saw a significant cytotoxic effect with the viability test. This study investigated the long-term effects after just 1 week for isolated cells, and the effect on tendon tissue after a longer time period might be of interest. Because no long-term effects on the cells were seen and the clinical data revealed no impaired healing or higher re-rupture rates, no long-term effects on the tissue level are expected.

## 5. Conclusions

The results of the present comprehensive study, showing no negative effects of vancomycin at different concentrations on the tendon structural properties and cell viability, support the existing in vitro data and the in vivo healing data as well as the clinical findings regarding safety. Even concentrations higher than the usual clinical use had no negative effects after 20 min of incubation. Although randomized prospective trials are lacking, the data from previous studies and this comprehensive analysis are in favor of this treatment as an infection prophylaxis in ACLR. The comprehensive approach utilized in this study may also be useful in other areas of tendon and ligament research to test the effects of drugs or treatments on tendons, ligaments and cells.

## Data Availability

All data are shown in the manuscript. Further information is available on request.
